# Overview of research activities associated with the World Health Organization: results of a survey covering 2006/07

**DOI:** 10.1186/1478-4505-8-25

**Published:** 2010-09-06

**Authors:** Robert F Terry, Tess van der Rijt

**Affiliations:** 1 Research Policy and Cooperation Department, World Health Organization, 1272 Geneva, Switzerland

## Abstract

**Background:**

This paper presents the first comprehensive effort to provide an overview of the research associated with the World Health Organization (WHO) headquarters in 2006/07.

**Methods:**

Information was obtained by questionnaire and interviews with senior staff operating at WHO headquarters in Geneva. Research type, purpose and resources (both financial and staff) were defined and compared for each of the 37 departments identified and a comparative analysis was made with the global burden of disease as expressed by Disability Adjusted Life Years (DALY).

**Results:**

Research expenditure in 2006/07 was estimated at US$215 million. WHO is involved in more than 60 research networks/partnerships and often WHO itself is the network host.

Using the DALY model, 84% of the funding WHO allocates to research goes to DALY Type I diseases (communicable, maternal, perinatal and nutritional diseases) which represents 40% of DALY. 4% is allocated to Daly Type II (non-communicable diseases) which contributes to 48% of DALY.

45% of WHO permanent staff are involved with health research and the WHO's approach to research is predominantly focused on policy, advocacy, health systems and population based research. The Organization principally undertakes secondary research using published data and commissions others to conduct this work through contracts or research grants. This approach is broadly in line with the stated strategy of the Organization.

**Conclusions:**

The difficulty in undertaking this survey highlights the complexity of obtaining an Organization-wide assessment of research activity in the absence of common standards for research classification, methods for priority setting and a mechanism across WHO, or within the governance of global health research more generally, for managing a research portfolio.

This paper presents a strategic birds-eye view of the WHO research portfolio using methodologies that, with further development, may provide the strategic information required if there is to be balancing of research efforts between communicable disease, non-communicable disease and other pressing public health needs. As the rollout of the WHO strategy on research for health proceeds we would hope to see similar exercises undertaken at the WHO Regional Offices and in support of capacity building of national health research systems within Member States.

## Background

In May 2010 the World Health Assembly adopted a resolution (WHA63.21) setting out WHO's roles and responsibilities in health research and endorsing the WHO Strategy on Research for Health [1]. One element of the strategy was to provide an overview of the research activities WHO is associated with. This report outlines the findings of the first overview in order to provide a measure of WHO's organizational role in supporting primary and secondary research and research capacity building; and to facilitate a comparative analysis of research activity between programmes.

## Methods

### Acronyms

The acronyms used in the text are defined in Table 1. The term 'department' is used for convenience as a collective term to describe an operational unit at WHO. These operational units or departments can be organized as programmes, partnerships or alliances. It is important to note the range in size of these operational units from 1-2 staff undertaking research within a WHO programme to departments where research is the primary focus.

**Table 1:** Research at WHO - data provided for the biennium budget 2006/07

Research Area	WHO Department	Acronym	^§^Global DALY Type	Research Expenditure ($US)	Number of projects supported	*Staff	
						
						Involved in research	Total staff
HIV/AIDs, TB, Malaria and Neglected Tropical Diseases	HIV/AIDS	HIV/AIDS	I	4,500,000	n/a	31	57

	Global Malaria Programme	GMP	I	1,350,000	n/a	5	23

	Stop TB Department	STB	I	9,100,000	n/a	25	114

	Control of Neglected Tropical Diseases	NTD	I	1,300,000	n/a	10	31

**Subtotal**				**63,350,000**	**n/a**	**71**	**225**

Health System and Services	Alliance for Health Policy & Systems Research	Alliance HPSR	IV	3,540,000	30	4	4

	Health System Governance & Service Delivery	HDS	IV	1,006,800	n/a	6	20

	Health System Financing	HSF	IV	172,000	n/a	4	19

	World Alliance for Patient Safety programme	PSP	IV	500,000	27	5	17

	Human Resources for Health	HRH	IV	1,500,000	n/a	5	16

	Essential Health Technologies	EHT	IV	100,000	6	2	19

	Essential Medicines & Pharmaceutical Policies	EMP	IV	1,600,000	n/a	40	85

**Subtotal**				**8,418,800**	**63**	**66**	**180**

Health Actions in Crisis	Emergency Preparedness and Capacity Building	EPC	III	5,600,000	n/a	3	4

	Emergency Response and Operations	ERO	III	100,000	n/a	4	11

	Recovery and Transition Programmes	REC	III	N/A	n/a	6	17

**Subtotal**				**5,700,000**	**n/a**	**13**	**32**

Information, Evidence and Research	Health Statistics and Informatics	HIS	IV	2,000,000	1	5	34

	Research Policy and Cooperation	RPC	IV	917,012	0	4	4

	Ethics, Equity, Trade & Human Rights	ETH	IV	30,000	6	4	21

	Health Metrics Network	HMN	IV	N/A	6	13	13

	Knowledge Management and Sharing	KMS	IV	0	0	0	64

**Subtotal**				**2,947,012**	**13**	**26**	**136**

							

Family and Community Health	Child & Adolescent Health & Development	CAH	I	5,600,000	39	16	26

	Gender, Women and Health	GWH	I	598,891	n/a	4	5

	Immunization, Vaccines and Biologicals	IVB	I	5,000,000	n/a	18	73

	Reproductive Health & Research	RHR	I	32,000,000	35	36	62

	Making Pregnancy Safer	MPS	I	1,000,000	n/a	7	20

	Initiative for Vaccine Research	IVR	I	20,000,000	25	17	18

	Polio Eradication Initiative	POL	I	2,500,000	20	11	38

**Subtotal**				**66,698,891**	**119**	**109**	**242**

Noncommunicable Diseases and Mental Health	Chronic Diseases and Health Promotion	CHP	II	4,000,000	27	10	80

	Violence & Injury Prevention & Disability	VIP	III	715,000	3	10	16

	Mental Health & Substance Abuse	MHS	II	15,000	2	4	15

	Nutrition for Health & Development	NHD	I	1,000,000	4	7	18

	Tobacco Free Initiative	TFI	II	2,000,000	n/a	17	35

	WHO Centre for Health and Development, Kobe	WHO Kobe Centre	IV	2,400,000	6	6	10

**Subtotal**				**10,130,000**	**42**	**54**	**174**

Health Security and Environment	Epidemic and Pandemic Alert Response	EPR	I	N/A	32	30	107

	Food Safety, Zoonoses & Food borne Diseases	FOS	I	400,000	17	8	15

	Protection of the Human Environment	PHE	I	8,460,000	75	32	44

**Subtotal**				**8,860,000**	**124**	**70**	**166**

	Special Programme for Research and Training in Tropical Diseases	TDR	I	44,600,000	642	38	43

	International Agency for Research on Cancer	IARC	II	51,400,000	210	102	115

**TOTAL**				**215,004, 703** **(n = 34)**	**1213** **(n = 22)**	**549** **(n = 37)**	**1313** **(n = 37)**

§ Global DALY Types: I Communicable disease, maternal, perinatal and nutritional diseases; II: Noncommunicable diseases; III: Injuries, war and violence. Type IV: Capacity is not a DALY Type and defined by the authors within this paper.

* Source: Questionnaire response plus WHO Staff Directory includes temporary and fixed term staff and excludes interns collated May 2008.

### Definitions of Research

After consultation with WHO departments and staff, the WHO Strategy on Research for Health defined research as the development of knowledge with the aim of understanding health challenges and mounting an improved response to them. This definition covers a spectrum of research, which spans five generic areas of activity: measuring the problem; understanding its cause(s); elaborating solutions; translating the solutions or evidence into policy, practice and products; and evaluating the effectiveness of solutions.

In each of these generic areas we used the term primary and secondary research where secondary research is research that uses existing data through analysis and/or synthesis and primary research is activity that generates new - primary - data. There is no intended hierarchy in the terms; both are essential activities to generate new knowledge.

The term "research WHO is associated with" was carefully chosen as it covers the different types of interaction WHO has with research. These associations include research that WHO manages directly defined as conducted research (often by WHO staff) and the research WHO commissions others to undertake. Commissioned research includes: contract research where the research question is defined by WHO; and the research that WHO manages indirectly following open competition, either funding through grants to individuals or institutions or fellowships. In addition there are situations where the Organization plays a technical or advisory role in guiding the research often as part of a network or partnership. Finally this definition also covers those activities concerned with research stewardship functions, policy creation and advocacy.

The focus of the research areas were categorized further using the following definitions agreed with the departments:

#### Basic science research

laboratory based, molecular or genetic, for example vaccine development.

#### Clinical

health research involving human participants most typically as a clinical trial. This is defined within WHO as any research study that prospectively assigns human participants or groups of humans to one or more health-related interventions to evaluate the effects on health outcomes. Interventions include but are not restricted to drugs, cells and other biological products, surgical procedures, radiological procedures, devices, behavioural treatments, process-of-care changes, preventive care, etc.

#### Health services/health systems research

examination of recipients of care, health financing and administration, health service delivery and the structure of health care systems.

#### Population based research

the examination of individuals within a larger scale, examining the social determinants of health, looking at the impact of environment on health, larger community-based research and cohort studies.

#### Policy and advocacy

research to understand the transformation of evidence into practice, how evidence can best be used to improve public health.

For the purposes of this report the activity of routine collection of data for surveillance was not included as a research activity. The use of such data for research purposes is included.

### Survey questionnaire and other sources of information

The survey questionnaire was completed by 35 WHO departments based at the Geneva headquarters and two major research institutes where WHO has direct involvement in the governance arrangements: the Kobe Centre for Health and Development in Japan and the International Agency for Research on Cancer (IARC) in France. The surveys were completed during 2008 and follow up work continued until May 2009. Each department provided answers to cover the two year budget (biennium) for 2006/07. WHO regional offices and collaborating centres were not covered by the survey.

The information collected in the questionnaire was supplemented with published reports, information taken from the central financial and administrative databases of WHO and by conducting interviews with the Director of each department or senior members of staff nominated by them.

### Expenditure and Sources of funding (Figures 1 and 2)

**Figure 1:**
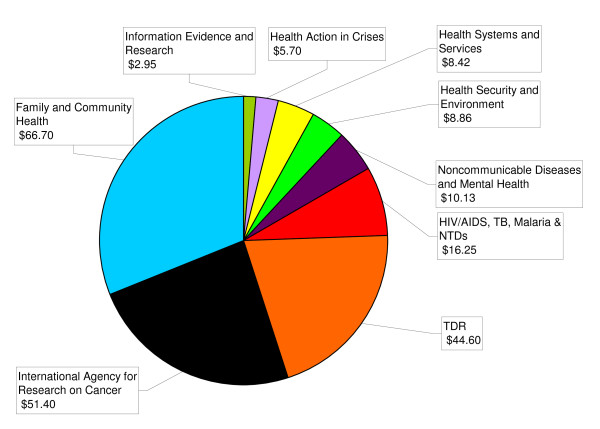
**WHO Health Research Expenditure $USD millions 2006/07 (n = 34)**.

**Figure 2:**
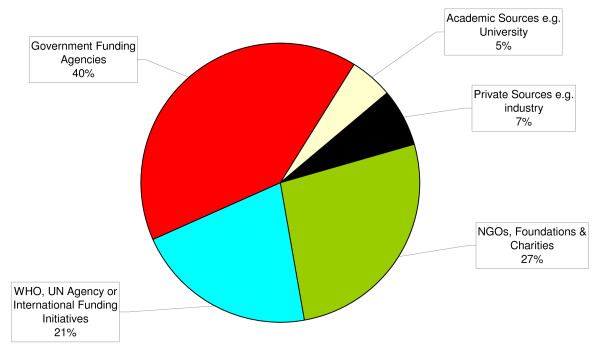
**Sources of research funds utilized by WHO 2006/07 (n = 37)**.

The data was compiled from the sources described above.

### The Radar diagrams - spider graphs (Figures 3 and 4)

**Figure 3:**
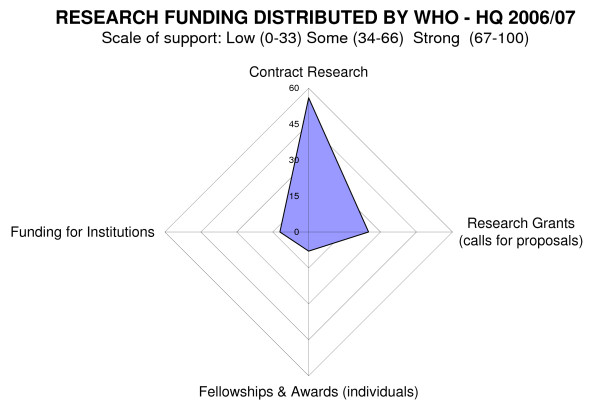
**Research funding distributed by WHO - HQ 2006/07**. Total $215 million (n = 35).

**Figure 4:**
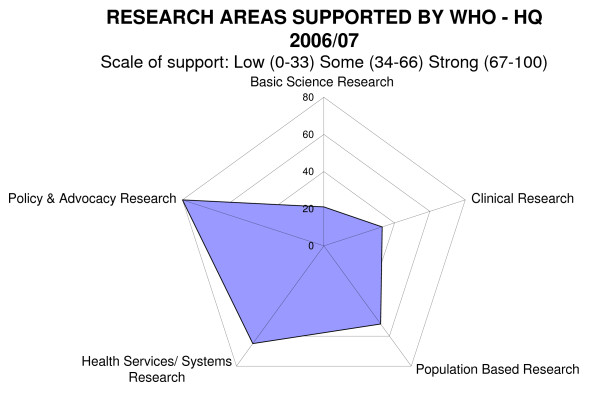
**Research areas supported by WHO-HQ 2006/07 (n = 35)**.

The Organizational radar diagrams were generated by asking the departments to provide an estimate of its scale of support (either low 1, some 2 or strong 3) in two areas: the type of approach used in funding research (contracted vs. commissioned) and the nature of the support across a range of defined research areas. These estimates were then aggregated across all the departments and normalised to a 0-100 scale where low support = 0 - 33; some support = 34 - 66 and strong support = 67 - 100. Therefore, a score of over 67 represents the majority of the departments reporting strong support in this area. The diagram affords equal weight to each departmental response, so it is important to note the radar diagram provides a visual representation of the current strategy taken by the Organization as a whole. It does not quantify the output of that approach; it does not include a weighting measure such as dispersion of research funds. Furthermore, a department could record strong support in more than one area as the areas are not mutually exclusive.

### The strategic shape of research at WHO the 'bubble' diagram (Figure 5)

**Figure 5:**
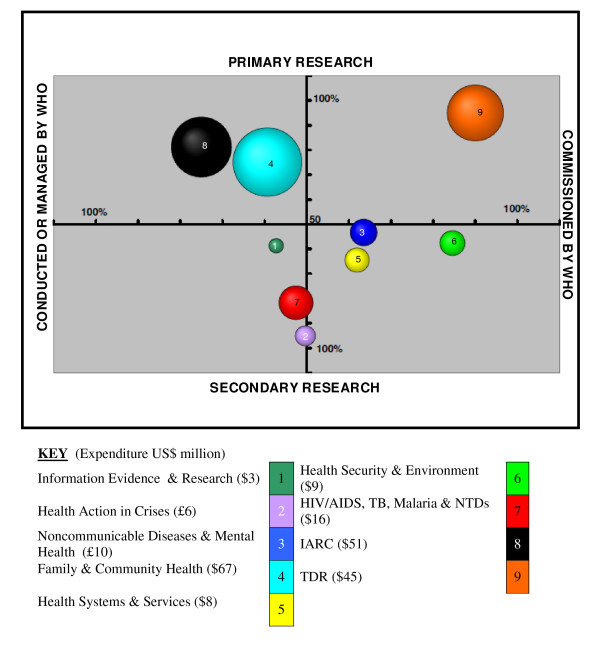
**Overview of Research at WHO 2006/07 (n = 36)**.

Departments were asked to estimate in percentage terms the degree to which the research they were associated with was either primary or secondary research (x-axis) and further to define whether they were conducting or commissioning the research (y-axis). The results were then grouped into research areas and plotted against these two axes taking the higher percentage figure in each set as the coordinates. The diameter of the bubble is in proportion to the research funds it dispersed in 2006/07.

### Comparing the research WHO is associated with against the global burden of disease estimated by disability adjusted life years 2004 - DALYs (Figure 6) [2]

**Figure 6:**
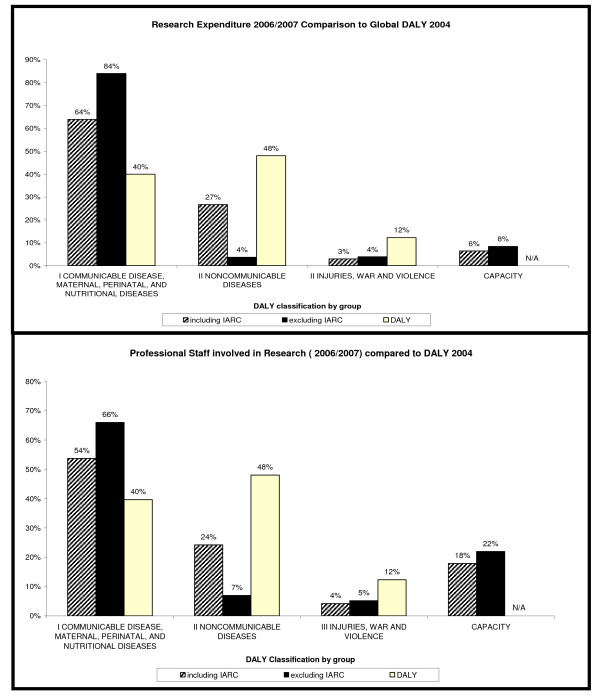
**Comparison of WHO -HQ Research with DALYs**.

In order to undertake this comparison we followed the method outlined by *Stuckler et al *in their paper on WHO's budgetary allocations and the estimates of the global burden of disease [3]. We grouped the research WHO is associated with into four categories: type I infectious disease; type II non-communicable diseases and type III injuries and violence. These correspond to the WHO disease and injury DALY types. There were also a number of departments that conduct research for which there is no corresponding estimate of DALYs. We called this group 'capacity' and this includes the departments working on health and research policy and systems, the health workforce, health financing, essential medicines and technologies, public health and patient safety.

To quantify the WHO response in each category, we compared the DALYs with the percentage of funding and the percentage of professional staff allocated to that area. Results were presented both including and excluding IARC, as this represents a large proportion of resource dedicated to cancer and tended to obscure the findings in other areas.

### Limitations of this report

All of the information obtained for the overview is that reported by the departments themselves and while clarification was sought on a number of points it is without independent verification. Many of the departments' responses are subjective and are qualitative judgements made by one or two senior individuals within a department. Efforts were made to standardize the responses across the departments, but it is important to recognize there is a large range in the degree of research activity within WHO, from those departments with a dedicated research focus dispersing millions of dollars, e.g. RHR/HRP and TDR, to departments where research is only one element of their total workplan.

In many programmes research activities are embedded within operational activities and the boundary between research and operation activities is difficult to disaggregate. As a result a number of departments were unable to exactly quantify the specific number of 'research projects' but counted projects which contained an element of research. For similar reasons no attempt was made to describe, at a departmental level, the research funded through core budget provided by Member States and that funded by extra-budgetary sources, Member States and other donors. These limitations remain areas for improvement in future overviews.

## Results and discussion

A questionnaire response was obtained from all 37 departments covered by the survey 100% (Table 1. n = 37). However, the response rate for individual questions varied and this is noted in the text.

Of the 37 departments surveyed 36 reported an element of research activity. KMS which includes the library, eHealth initiatives and WHO Press, did not report any research activities. The two research centres (IARC and Kobe) and 5 of the departments (Alliance HPSR, IVR, RHR, RPC and TDR) report that research is their primary activity. Of the staff included in the survey, 45% (549 out of a total 1208) are reported as being involved in research under the strategy's definition with 13 departments having more than half their staff involved in research.

The total amount of research expenditure in 2006/07 (n = 34) was US$215 million. Excluding the two research centres, the total was US$161 million (Figure 1). Figures were not available from Epidemic & Pandemic Alert & Response, Recovery & Transition Programmes and the Health Metrics Network. Within headquarters (excluding IARC and Kobe) more than half of this funding is dispersed by 3 departments: the Initiative for Vaccine Research; Reproductive Health and Research within the Family and Community Health cluster; and the Special Programme for Training and Disease Research (TDR). These 3 departments contain 21% of the staff involved in research.

The remaining departments at headquarters (n = 29) each reported expenditure of US$9 million or less, with 13 reporting individual expenditure of under US$1 million. However, it is worth noting these 29 departments contain nearly 80% of the staff reported to be involved with research.

### The source of funds

The findings are that 20% of funding for the research that WHO is associated with comes from within the UN system. As such, 80% is from other sources, the main two being government funding agencies (40%) and NGOs, foundations and charities (27%). The remaining sources are private (industry) 7% and academia 5% (Figure 2).

The 80% of funding that is not from within the UN system is considered voluntary contributions, which the departments often apply for. Therefore, the majority of research funds WHO disperses are designated funding that are earmarked for specific projects creating a close association between the donors that support WHO and the research WHO supports.

### Partnerships

While the total sum of money WHO distributes is modest compared to global figures, WHO rarely operates in isolation and every department reported that its work was often undertaken as part of an alliance, partnership or research network. The survey found that WHO is involved in more than 60 networks and often WHO itself is the network host. The survey does not measure the leverage effect of WHO funding, but this finding suggests it is considerable.

### How the research money is distributed

WHO has two main approaches to the distribution of research funding. Research is commissioned through contracts or distributed as grants through open proposals to projects, to institutions or individuals as fellowships or travel awards (Figure 3).

The figure shows that, for the Organization as a whole, the main funding approaches are for commissioned research contracts and research grants awarded in response to an open call. Funding for individuals is only reported as strong by the IARC and a limited number of fellowships were available through RHR and TDR.

### Research areas supported by WHO

Departments described the degree of support they have for 5 different research areas: basic science, clinical, health services/systems, population based, policy and advocacy. This information was aggregated to provide an overview of the Organization's research areas (Figure 4).

This diagram illustrates that the approach towards research at an Organizational level is focused on policy and advocacy, health services and systems research and population based research. Considering the mission of the Organization, this might be expected.

Basic and clinical research tends to be supported in those departments with research as a major activity or primary focus and includes TDR, IARC, RHR, IVR CAH, NTD, FOS and GMP. It is in the basic and clinical areas where the majority of resources to support research are spent.

### The strategic overview of research at WHO the 'bubble' diagram (Figure 5)

Figure 5 represents the research approach taken by WHO in relation to primary and secondary research, plotted against whether the research is directly managed by WHO or commissioned, i.e. undertaken by others. The diagram provides a visual representation to enable a discussion of the strategic organizational approach as a whole. It poses the question: should the Organization be evenly spread across all quadrants of the graph or concentrated in one area e.g. the bottom right hand corner - primarily commissioning secondary research?

In 2006/07, WHO research is found in all four quadrants, showing there is support across primary and secondary research that is both commissioned by the Organization and managed by it. The majority of the departments are shown as having a higher percentage of support for secondary research.

### Comparing the research WHO is associated with against the global burden of disease estimated by disability adjusted life years for 2004

Comparing research expenditure 2006/07 to the DALYs for 2004 (Figure 6) shows that, when IARC is excluded, 84% of WHO's research budget is allocated to type I diseases that accounts for 40% of estimated DALYs. 4% of the research budget is allocated to type II Non-communicable disease that accounts for 48% of estimated DALYs. 4% of the research budget is allocated to type III Injuries, war and violence that accounts for 12% of estimated DALYs. In capacity (our definition) 8% of the budget is allocated, with no estimation for DALYs.

When a comparison is made of the number of staff allocated to research in these DALYs, a similar pattern is evident, with a greater proportion of staff associated with research in DALY type I diseases (66% excluding IARC) than in type II (7% excluding IARC).

### Geographical spread of research projects

For many departments, research is not a discrete activity but is embedded within the programmes. When asked to identify the number of separate research projects the departments were supporting this proved difficult to obtain. There were 22 departments that provided an estimated figure, which identified 1213 supported projects. In this sample 92% of the projects are operational outside of OECD countries (Table 2).

**Table 2:** Number of projects and geographical spread

	Total
Number of projects supported	1213 (n = 22)^a^

Number of projects led by a Principal Investigator from an OECD country	108 in 299 projects (n = 13)^b^

Number of projects operational in an OECD country	24 in 299 Projects (n = 11) ^b^

a Includes IARC but data for 15 departments was not available

b Excludes IARC

Of the departments that responded, 13 reported that the principal investigator was from an OECD country in 108 (36%) projects out of a total of 299 supported by these 13 departments, and 24 of these projects were based in an OECD country (8%).

The figures collected are an underrepresentation of current activity but indicate that the majority of WHO support for research is in the middle and low income countries, led by researchers based in those countries.

## Conclusions

In varying degrees, research activity is found throughout the operational departments based at WHO headquarters and nearly half of the staff (45%) are involved in research activities as defined here.

When asking staff for their opinion, the strategic 'shape' of the organizational-level approach to research is focused on policy, advocacy, health systems and population based research. WHO principally undertakes secondary research using published data and commissions others to undertake this work through contracts or research grants. This approach is broadly in line with the stated strategy of the Organization as a whole. These departments work within over 60 research networks. This suggests that while WHO funds for supporting research are modest compared to global resources, the leverage effect of being an active partner in the research effort is considerable.

When research activity is assessed by examining resources, the majority of the research budget WHO disperses is funding primary research in basic and clinical areas, product development and vaccine initiatives. Using the DALY model, 84% of the funding WHO allocates to research goes to Type I diseases (communicable, maternal, perinatal and nutritional diseases) which represents 40% of DALY. Only 4% of funding is allocated to Type II (non-communicable diseases) which contributes to 48% of DALY. This unequal spread of resources might be a reflection that basic and clinical research in the Type I diseases is disproportionately more expensive than that for the Type II area. However, the picture is the same for the number of staff allocated to research in this area and tends to support the findings of others that the allocation of WHO resources is skewed towards infectious disease [3]. This situation is compounded by the observation that the majority of funds WHO disperses (80%) is designated funding earmarked for specific projects. This means there is a close association between the goals of the donors that support WHO and the research WHO supports.

These conclusions are similar to those previously identified in a less comprehensive study conducted by Sida in 2005 and by stakeholders consulted during the development of the WHO Strategy on Research for Health [1,4]. Within that strategy, the Goal focusing on the Organization seeks to address these issues where it states that WHO working with Member States and partners will:

'...establish appropriate structures for keeping abreast of latest developments in knowledge management, interaction with the global research community, and leading, managing and coordinating research within WHO, and for maintaining accountability for such research; and secure the resources needed to support the implementation and evaluation of this strategy.'

The implementation of the strategy will provide a strong opportunity to build on the findings presented here and to institutionalize the regular collection, analysis and communication of such data and ultimately translate research evidence into practice that has a measureable impact on improving health.

The difficulty in undertaking this survey and the length of time required to extract these data highlights the complexity of obtaining an organization-wide assessment of research activity. This situation is reflective of efforts in global health more generally, the governance of which has been described as chaotic [5]. There are no common standards for research classification, methods for priority setting and no mechanism across WHO, or within the governance of global health research more generally, for managing a research portfolio as a whole. More importantly if there is to be greater harmonization of global research activities, as called for by Health Ministers in Bamako in 2008, more work will need to be done in identifying such common approaches to allow for comparable data and benchmarks to manage, organize or evaluate the global research portfolio [6]. This paper presents a birds-eye view using methodologies that, with further development, may assist with evaluations of that kind in the future. It should also provide the strategic information required if there is to be balancing of research efforts between communicable disease, non-communicable disease and other pressing public health needs. As the rollout of the WHO strategy on research for health proceeds we would hope to see similar exercises undertaken at the WHO Regional Offices and in support of capacity building of national health research systems within Member States.

## Competing interests

The Authors declare no competing interests. The work was funded under a grant from the Bill and Melinda Gates Foundation (Number: 49275.01)

## Authors' contributions

RT conceived the study concept, design and is responsible for the main data analysis, presentation and conclusions. TvdR undertook the survey, initial analysis and first draft of the paper. All authors read and approved the final manuscript.
